# Ultrastructural insights into cellular organization, energy storage and ribosomal dynamics of an ammonia-oxidizing archaeon from oligotrophic oceans

**DOI:** 10.3389/fmicb.2024.1367658

**Published:** 2024-04-26

**Authors:** Yangkai Zhou, An Yan, Jiawen Yang, Wei He, Shuai Guo, Yifan Li, Jing Wu, Yanchao Dai, Xijiang Pan, Dongyu Cui, Olivier Pereira, Wenkai Teng, Ran Bi, Songze Chen, Lu Fan, Peiyi Wang, Yan Liao, Wei Qin, Sen-Fang Sui, Yuanqing Zhu, Chuanlun Zhang, Zheng Liu

**Affiliations:** ^1^Shenzhen Key Laboratory of Marine Archaea Geo-Omics, Department of Ocean Science and Engineering, Southern University of Science and Technology, Shenzhen, Guangdong, China; ^2^Cryo-Electron Microscopy Center, Southern University of Science and Technology, Shenzhen, Guangdong, China; ^3^Department of Biology, Southern University of Science and Technology, Shenzhen, Guangdong, China; ^4^Shanghai NanoPort, Thermo Fisher Scientific Inc., Shanghai, China; ^5^Institut AMU-WUT, Aix-Marseille Université and Wuhan University of Technology, Wuhan, Hubei, China; ^6^Australian Institute for Microbiology & Infection, University of Technology Sydney, Ultimo, NSW, Australia; ^7^School of Biological Sciences and Institute for Environmental Genomics, University of Oklahoma, Norman, OK, United States; ^8^State Key Laboratory of Membrane Biology, Beijing Frontier Research Center for Biological Structures, Beijing Advanced Innovation Center for Structural Biology, School of Life Sciences, Tsinghua University, Beijing, China; ^9^Shanghai Sheshan National Geophysical Observatory, Shanghai, China; ^10^Advanced Institute for Ocean Research, Southern University of Science and Technology, Shenzhen, Guangdong, China

**Keywords:** ammonia-oxidizing archaeon, *Nitrosopumilus maritimus* SCM1, cryo-electron tomography (cryo-ET), scanning transmission electron microscopy (STEM), energy dispersive X-ray spectroscopy (EDS)

## Abstract

**Introduction:**

Nitrososphaeria, formerly known as *Thaumarchaeota*, constitute a diverse and widespread group of ammonia-oxidizing archaea (AOA) inhabiting ubiquitously in marine and terrestrial environments, playing a pivotal role in global nitrogen cycling. Despite their importance in Earth’s ecosystems, the cellular organization of AOA remains largely unexplored, leading to a significant unanswered question of how the machinery of these organisms underpins metabolic functions.

**Methods:**

In this study, we combined spherical-chromatic-aberration-corrected cryo-electron tomography (cryo-ET), scanning transmission electron microscopy (STEM), and energy dispersive X-ray spectroscopy (EDS) to unveil the cellular organization and elemental composition of *Nitrosopumilus maritimus* SCM1, a representative member of marine *Nitrososphaeria*.

**Results and Discussion:**

Our tomograms show the native ultrastructural morphology of SCM1 and one to several dense storage granules in the cytoplasm. STEM-EDS analysis identifies two types of storage granules: one type is possibly composed of polyphosphate and the other polyhydroxyalkanoate. With precise measurements using cryo-ET, we observed low quantity and density of ribosomes in SCM1 cells, which are in alignment with the documented slow growth of AOA in laboratory cultures. Collectively, these findings provide visual evidence supporting the resilience of AOA in the vast oligotrophic marine environment.

## Introduction

Comprehending the intricate cellular structure of microorganisms holds paramount importance in advancing our understanding of metabolic functions in ecology, evolution, and biogeochemistry (González-Pech et al., 2023). Despite remarkable advancements in cryo-electron microscopy (cryo-EM) technology and the potential value of archaeal structural biology in medical and industrial applications (Shin et al., 2014), the realm of structural biology of marine archaea remains relatively underexplored, necessitating efforts to comprehensively grasp their cellular structure and physiological mechanisms (Lipp et al., 2008; Stahl and de la Torre, 2012).

Ammonia-oxidizing archaea (AOA) belong to the class *Nitrososphaeria*, previously named as the phylum *Thaumarchaeota* and *Nitrososphaerota* (Brochier-Armanet et al., 2008; Stieglmeier et al., 2014; Oren and Garrity, 2021; Rinke et al., 2021). They derive energy from the oxidation of ammonia to nitrite, initiating nitrification process that is vital in the aquatic and terrestrial nitrogen cycle (Treusch et al., 2005; Walker et al., 2010; Stahl and de la Torre, 2012). The resulting nitrite, a byproduct of AOA activity, can be further transformed into nitrate by nitrite-oxidizing bacteria, and ultimately eliminated from the ecosystem as it converts into nitrogen gas through denitrifying bacteria (Martikainen, 2022). Notably, *Nitrosopumilus maritimus* SCM1 stands out as the first isolate of AOA (Konneke et al., 2005), exemplifying one of the most prevalent ammonia-oxidizing microbes in the oceans and playing an active role in driving global nitrogen cycling.

Previous studies have delved into various aspects of *Nitrosopumilus maritimus* SCM1, including its physiology, such as ammonium oxidation, stress adaptation, and carbon fixation, as well as its genome, metabolome, lipidome, evolution and ecology (Martens-Habbena et al., 2009; Walker et al., 2010; Qin et al., 2014, 2018, 2020; Li et al., 2018; Kim et al., 2019; Kitzinger et al., 2019, 2020; Abby et al., 2020; Yang et al., 2021; Law et al., 2021a,b; Kraft et al., 2022; Hodgskiss et al., 2023; Leavitt et al., 2023; Wan et al., 2023). However, our knowledge of its cellular structure remains limited. Within an archaeal cell, the cellular components are organized in a specific arrangement. Starting from the cell surface and moving inward, these components typically include a surface layer (S-layer), pseudoperiplasmic space, cytoplasmic membrane, and the cytoplasm containing such as the nucleoid, ribosomes, and the enzymes involved in the cellular metabolism. Additionally, certain archaeal cells may possess additional structures like vesicles, archaella and their anchoring machinery, pili, and storage granules (van Wolferen et al., 2022). Several studies have reported the presence of S-layer proteins in the surface of SCM1 cells, suggesting its existence as a cell wall (Urakawa et al., 2011; Qin et al., 2017). Additionally, ribosome numbers of ~1,000 in each SCM1 cell were estimated, based on cryo-electron tomography (cryo-ET) data (Urakawa et al., 2011). The low number of ribosomes might provide an advantage in coping with extreme environmental conditions (Yin et al., 2018).

However, the cellular structure of AOA, including for example the presence and function of storage granules and the quantity of ribosomes, remains inadequately studied. Storage granules play a pivotal role in microorganisms by enabling them to withstand fluctuations in nutrient availability. Although extensive investigations have been conducted on storage granules in certain bacteria, thermophilic archaea, and eukaryotic organisms (Toso et al., 2011, 2016; Ward et al., 2012; Tocheva et al., 2013; Gal et al., 2017; Sarkar et al., 2021), their existence and functionality in marine archaea, particularly in SCM1, have yet to be thoroughly examined (Urakawa et al., 2011; Qin et al., 2017). This knowledge gap necessitates further exploration to unravel the cellular structure and evaluate the presence and function of storage granules in marine archaea, and thereby expanding our understanding in this uncharted domain.

This study aimed to address the knowledge gap in structural biology of AOA by employing techniques that combine spherical-chromatic-aberration-corrected cryo-EM, cryo-ET, scanning transmission electron microscopy (STEM), and energy-dispersive X-ray spectroscopy (EDS). These technologies allowed us to visualize the cell morphology of the AOA type strain *Nitrosopumilus maritimus* SCM1 *in situ*, which demonstrated the presence of two types of storage granules and putative cell division patterns, providing new insight into the potential metabolic functions of storage granules in marine archaea. This study utilized a new method of spherical-chromatic-aberration-corrected cryo-ET, which enabled a clear visualization of cellular organization within the three-dimensional (3D) volume of SCM1 cells that can extend up to 400 nm in thickness. This novel technology promises effective and comprehensive visualization not only of archaeal and bacterial cells but also of regions of eukaryotic cells up to 400 nm in thickness.

## Results

### Whole-cell cryo-electron tomography of *Nitrosopumilus maritimus* SCM1

We monitored cell growth in *Nitrosopumilus maritimus* SCM1 cultures by analyzing nitrite concentration. As shown in Supplementary Figure 1, during the 9 days’ cultivation period, nitrite concentration continued to rise in the first 7 days. We harvested SCM1 cells between day 6 and day 8, corresponding to the middle to late exponential growth phase. The purity of the cultures was evaluated using the qPCR method, which indicated bacterial contamination of less than 0.001% (Supplementary Table 1).

Initially, we examined the morphology of *Nitrosopumilus maritimus* SCM1 cells by cryo-EM. Our observations revealed that SCM1 cells showed a typical rod shape, with diameters ranging from 320 to 410 nm, lengths spanning from 590 to 1,350 nm and the mean length-diameter ratio around 2.43 ± 0.57 (Supplementary Figure 2). Subsequently, cryo-ET experiment elucidated the cellular organization of SCM1 cells and provided a native 3D structural information of the cell. Given the considerable thickness of the SCM1 cells (averaged diameter ~ 360 nm, Supplementary Figure 2), the tilt-series image acquisition could not be completed. The thickness is double at ±60°, rendering it difficult for cryo-EM data acquisition. To overcome this difficulty, we employed a spherical-chromatic-aberration-corrected cryo-electron microscope, which can increase the signal/noise ratio for thick specimen by correcting inelastic scattering. With the spherical-chromatic-aberration-corrected TEM, we were able to record datasets up to a tilt of ±60° on these specimens (Supplementary Figure 3), and tomograms were reconstructed with a smaller missing wedge.

**Figure 1:**
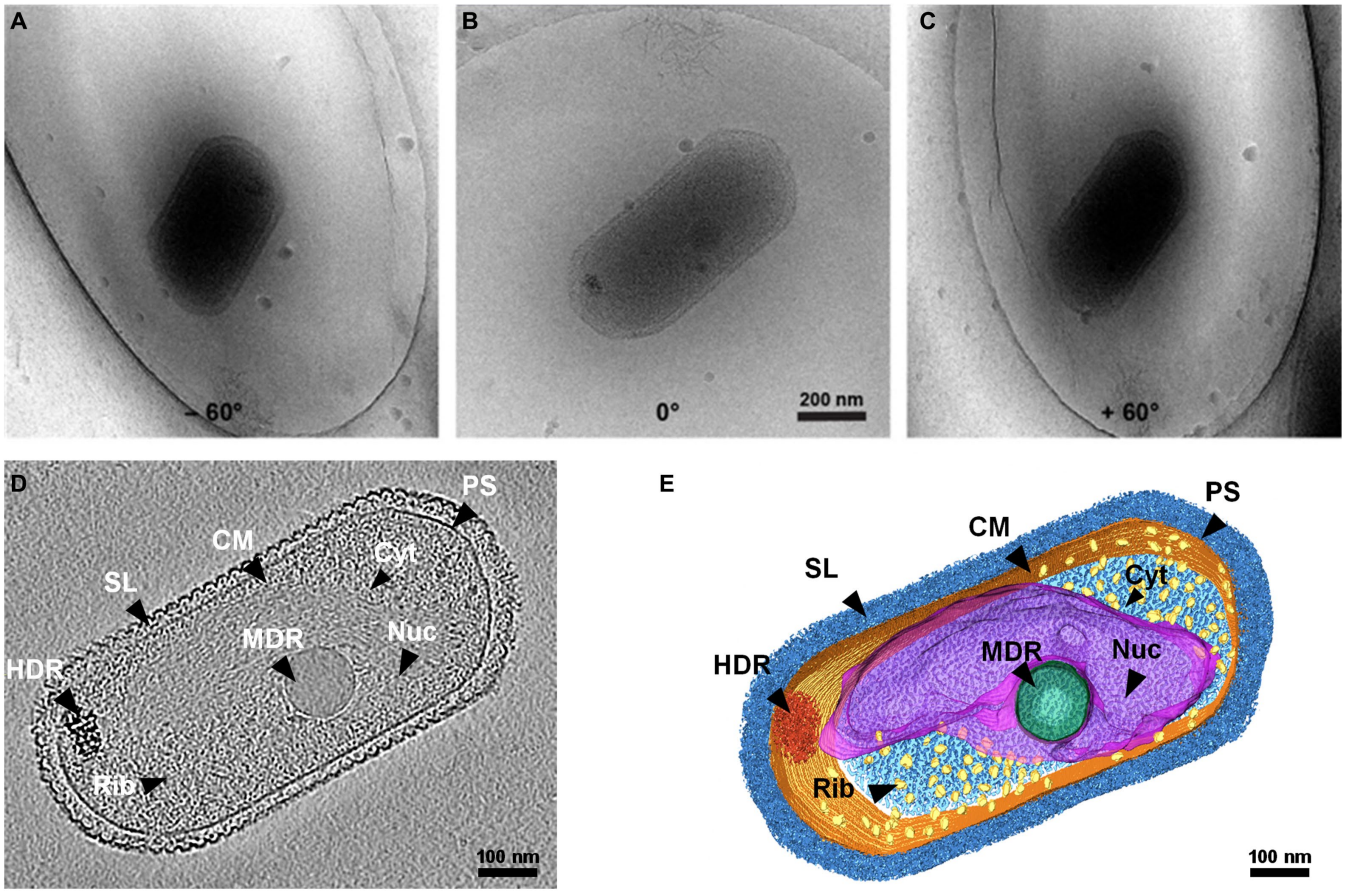
Whole-cell cryo-electron tomogram of *Nitrosopumilus maritimus* SCM1. **(A–C)** Cryo-EM images of SCM1 at − 60°, 0°, and + 60° angles. **(D)** Tomographic slice (13.6 Å in thickness) showed the cellular structure of SCM1. **(E)** 3D segmentation of SCM1 cell. MDR, medium-density region; HDR, high-density region; CM, cytoplasmic membrane; SL, surface layer; Nuc, nucleoid; Rib, ribosome; PS, pseudoperiplasmic space; and Cyt, cytoplasm, are displayed.

**Figure 2:**
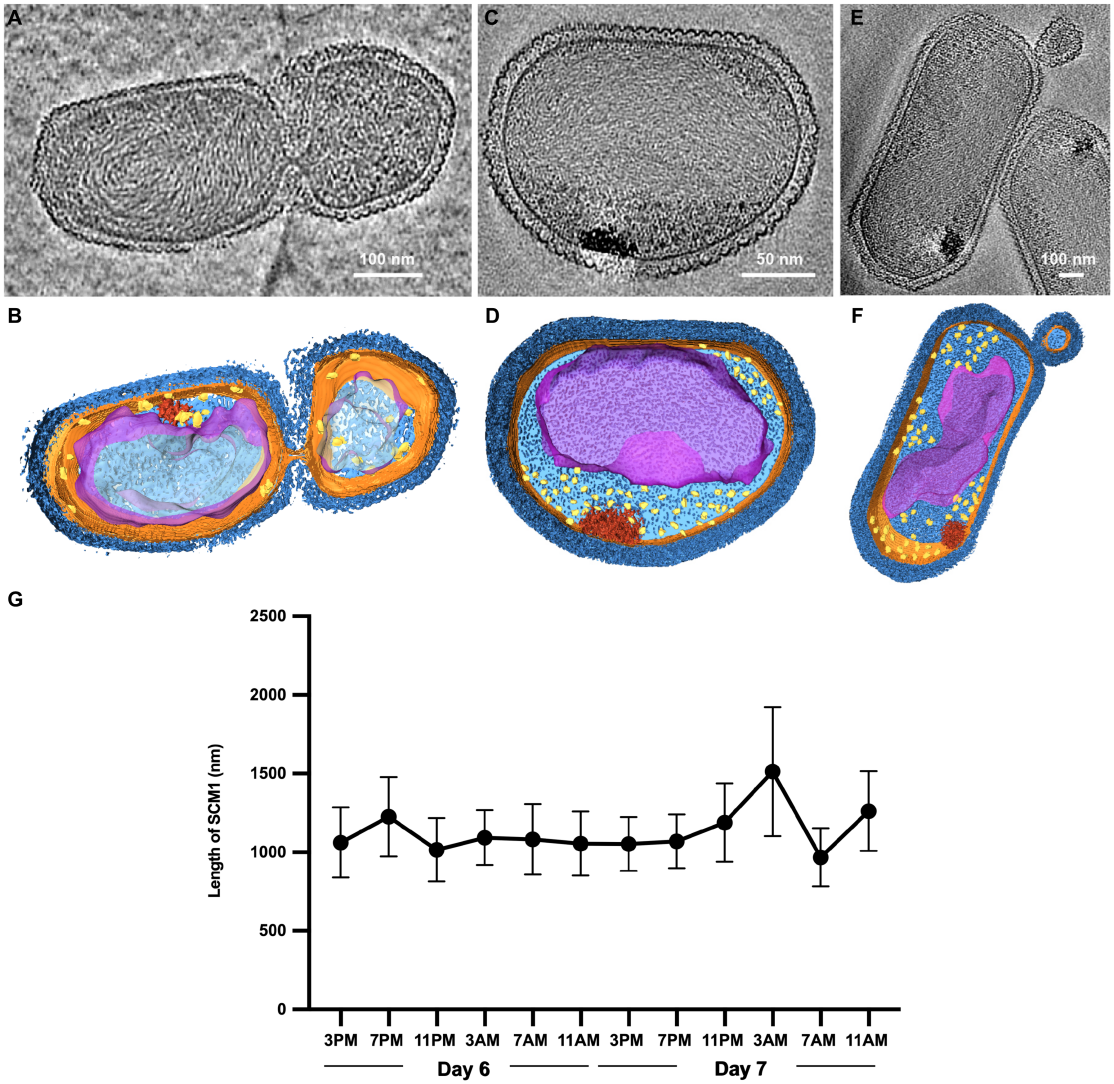
Observation of cell division in *Nitrosopumilus maritimus* SCM1. **(A,C,E)** Tomographic slices (13.6 Å in thickness) of SCM1 cells in the exponential phase. **(B,D,F)** 3D segmentation of **(A,C,E)**. S-layer (dark blue), cell membrane (orange); cytoplasm (light blue); nucleoid (purple); ribosomes (yellow); high-density region (red). **(G)** The average length of SCM1 at different time points. SCM1 cells were analyzed every 4 h for 48 h period in the late exponential phase (*n* = 40).

The 3D structure of native SCM1 revealed a highly organized proteinaceous S-layer enveloping the entire cell surface, and cell membrane exhibited well-defined, smooth, and continuous features (Figure 1 and Supplementary Movie 1). Notably, an area rich in nucleic acids and dozens of ribosomes were observed in the cytoplasm of SCM1 (Figures 1, 2).

### Observation of cell division in *Nitrosopumilus maritimus* SCM1

We examined several cells in the exponential phase and identified instances of unequal divisions (Figures 2A,B). The central region of SCM1 first exhibited constriction, followed by elongation toward two ends before eventually dividing into two cells. Notably, the cell division of SCM1 did not follow a homomorphic pattern, with the larger daughter cell assuming an oblate spheroidal shape, resembling the shape of another independently observed cell in Figures 2C,D. Additionally, we observed a putative budding state of SCM1 (Figures 2E,F).

Moreover, to determine the predominant mode of SCM1 cell division, we repeatedly measured the diameters and lengths of SCM1 in the late exponential phase. The results revealed two noticeable decreases in the length of SCM1 within a 48-h period, occurring at 11 PM on the Day 6 and 7 AM on the Day 7, respectively (Figure 2G). However, no notable change in diameter was observed within the 48-h timeframe. The time interval between the two decreasing points aligns with the doubling time of SCM1 (Td ≈ 30 h, Supplementary Figure 2), indicating a high level of synchronicity in cell division.

### Characterization of intracellular storage granules

Our cryo-ET results revealed density regions corresponding to granule in the cytoplasm of SCM1 (Figures 1D,E). Based on differences in electron density, we classified them into medium-density regions (MDR) and high-density regions (HDR). The MDR observed in SCM1 displayed a distinct and consistent spherical morphology, with diameters typically ranging from approximately 85–185 nm, with a smooth and continuous surface. The HDR exhibited an irregular shape and comprised varying electron density. Most HDRs were situated at the edge of cell, near the cell membrane (Figures 1, 2). In our datasets, we typically found one MDR and one HDR in each SCM1 cell; occasionally, we observed more than two MDRs in one cell.

**Figure 3:**
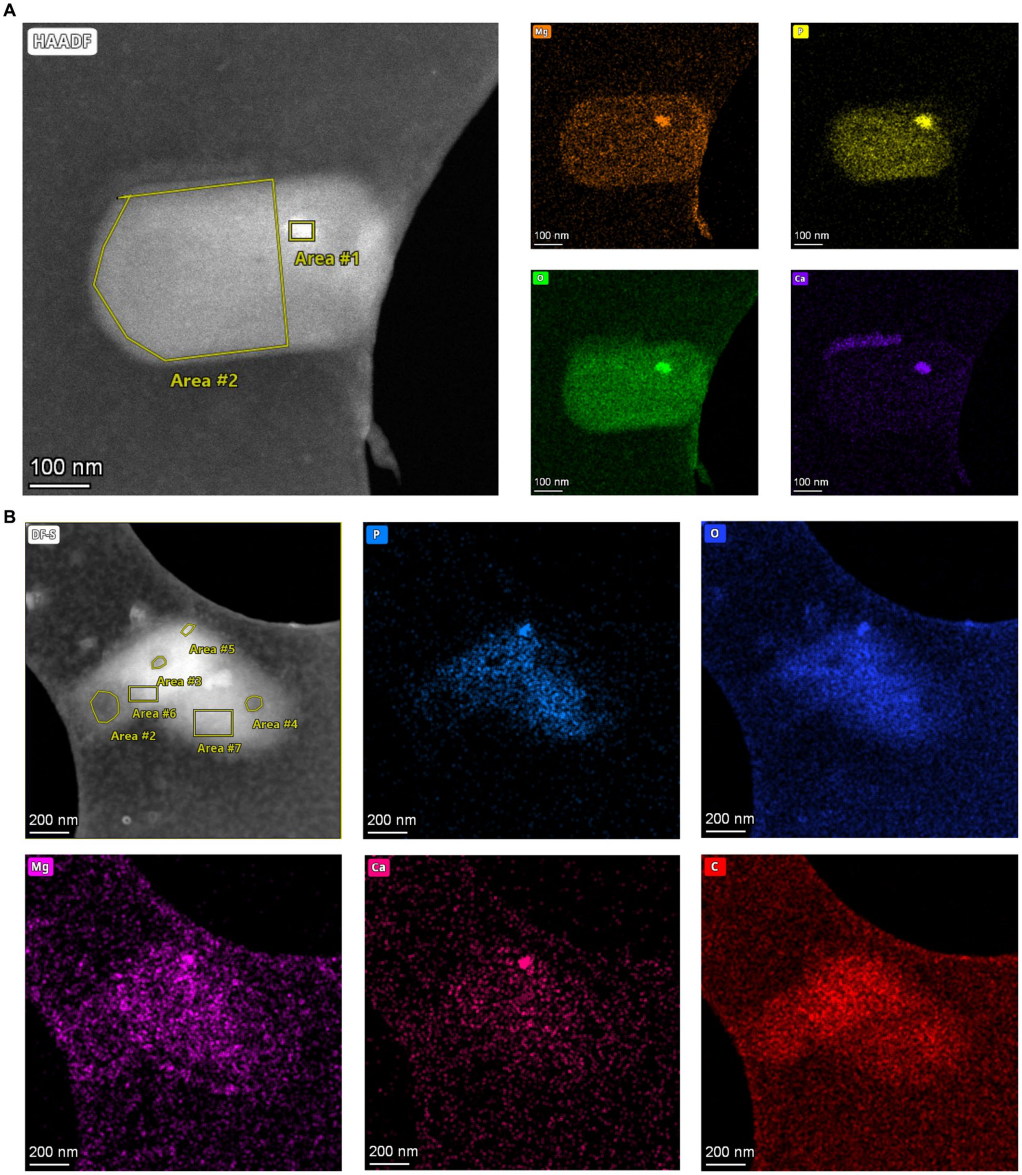
Elements in storage granules of SCM1. **(A)** STEM images and EDS elemental analysis revealed that magnesium (Mg), phosphorus (P), oxygen (O), and calcium (Ca) in the Area #1 stood out from the background, indicating that these elements were enriched in the HDR. **(B)** STEM-EDS results of two adjacent SCM1 cells. Area #5 is a HDR that is enriched with Mg, P, O, and *Ca.* Area #2, 3, and 4 are MDRs with slightly higher concentration of carbon (C).

To analyze the elemental composition of MDR and HDR granules in the SCM1 cells, we used a Talos F200X to obtain EDS spectra. Area scans revealed differential compositions of the granules: while HDRs containing elevated concentrations of calcium, phosphorus, oxygen, and magnesium, the MDRs had a slight increase in carbon concentration and a slightly lower concentration of oxygen (Figure 3 and Supplementary Table 2). Carbon and nitrogen were the most abundant elements throughout the entire scanned region across the cells.

**Figure 4:**
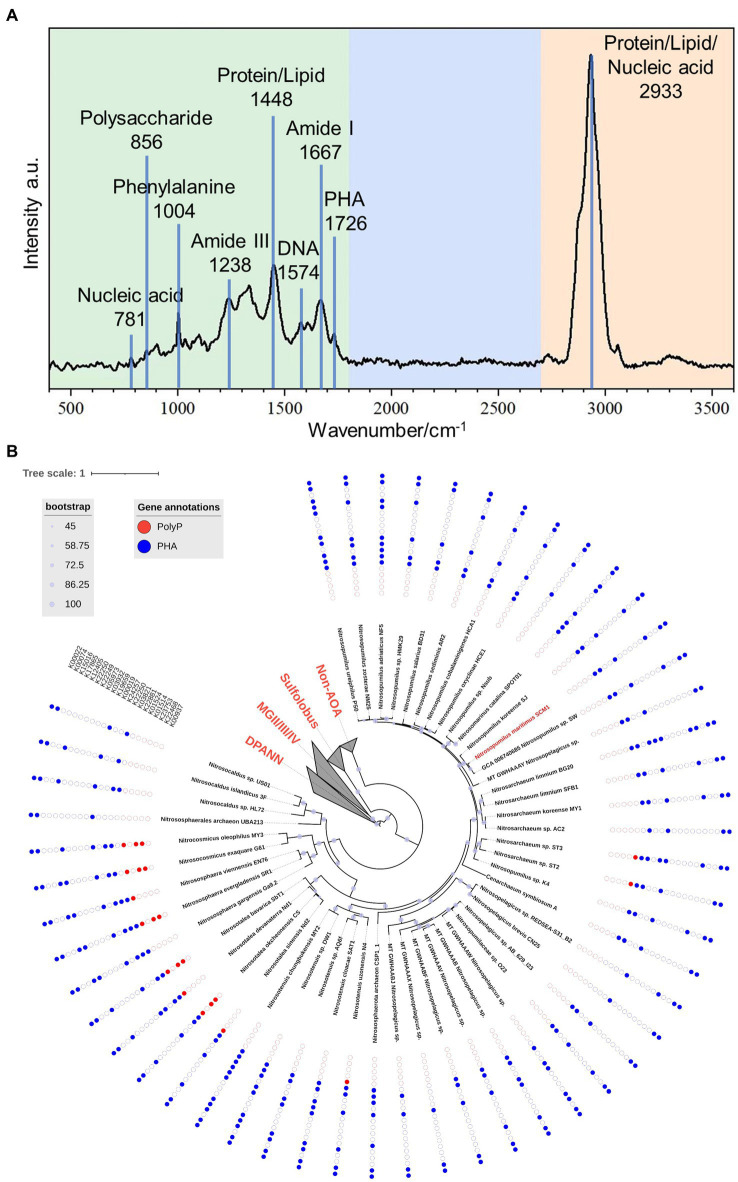
The Raman spectrum of *Nitrosopumilus maritimus* SCM1 and the distributions of metabolic genes regarding PolyP and PHA in AOA. **(A)** The mean Raman Spectrum of 33 single cells of SCM1. A characteristic peak of PHA at 1726 cm^−1^ was identified. **(B)** The phylogenomic tree of 51 nonredundant AOA genomes. The presence or absence of metabolic genes related to PolyP and PHA annotated by KEGG are shown around the tree. Solid dots: presence of the gene, empty dots: absence of the gene.

The mean Raman spectrum of SCM1 showed main biomolecules in single cells (Figure 4A). The Raman band at 1726 cm^−1^, which was slightly shifted from 1739 cm^−1^ in *Nitrososphaera gargensis* (Spang et al., 2012), could be attributed to polyhydroxyalkanoate (PHA). This shift of band was caused by the stretching vibration of the C=O ester in PHA (Ciobotă et al., 2010).

To further assess the metabolic potential of *Nitrosopumilus maritimus* SCM1 in utilizing storage granules and gain insights into the metabolic capabilities across higher taxa, we conducted an in-depth exploration of key metabolic pathways in *Nitrosopumilus maritimus* SCM1 and other AOA genomes, focusing on Polyphosphate (PolyP) and polyhydroxyalkanoate (PHA) metabolism. After conducting a thorough genome annotation, we identified 6 PHA metabolic genes in SCM1, including K22881 (*PhaE*), K03821 (*PhaC*), K00019 (*BdhA*), K12405 (hydratase), K00074 (dehydrogenase), and K00022 (dehydrogenase). PHA synthesis in SCM1 is primarily conducted by *PhaE* and *PhaC*, while *BdhA*, along with one hydratase (K12405) and two dehydrogenases (K00074 and K00022), is involved in PHA degradation. The proteome data confirmed the presence of all these proteins (Supplementary Table 3). However, despite the presence of intracellular PolyP confirmed by STEM-EDS analysis, we found no homologous genes associated with PolyP synthesis and degradation, suggesting additional undiscovered genes involved in PolyP metabolism in SCM1.

In other AOAs, we observed the presence of PolyP and PHA metabolic genes across various strains (Figure 4B). Notably, PolyP genes were identified in AOA strains ST2, ST3, N4, Nd1, Nd2, CS, SbT1, SR1, EN76, G61, and MY3. Among these strains, only ST2 and ST3 were found in marine habitats, specifically wastewater treatment beside the ocean. The occurrence of *Ppk2* genes in terrestrial AOA strains (CS, SbT1, SR1, G61, MY3) highlighted the ecological diversity and metabolic versatility within the AOA clade.

### Distribution of ribosomes in SCM1

**Figure 5:**
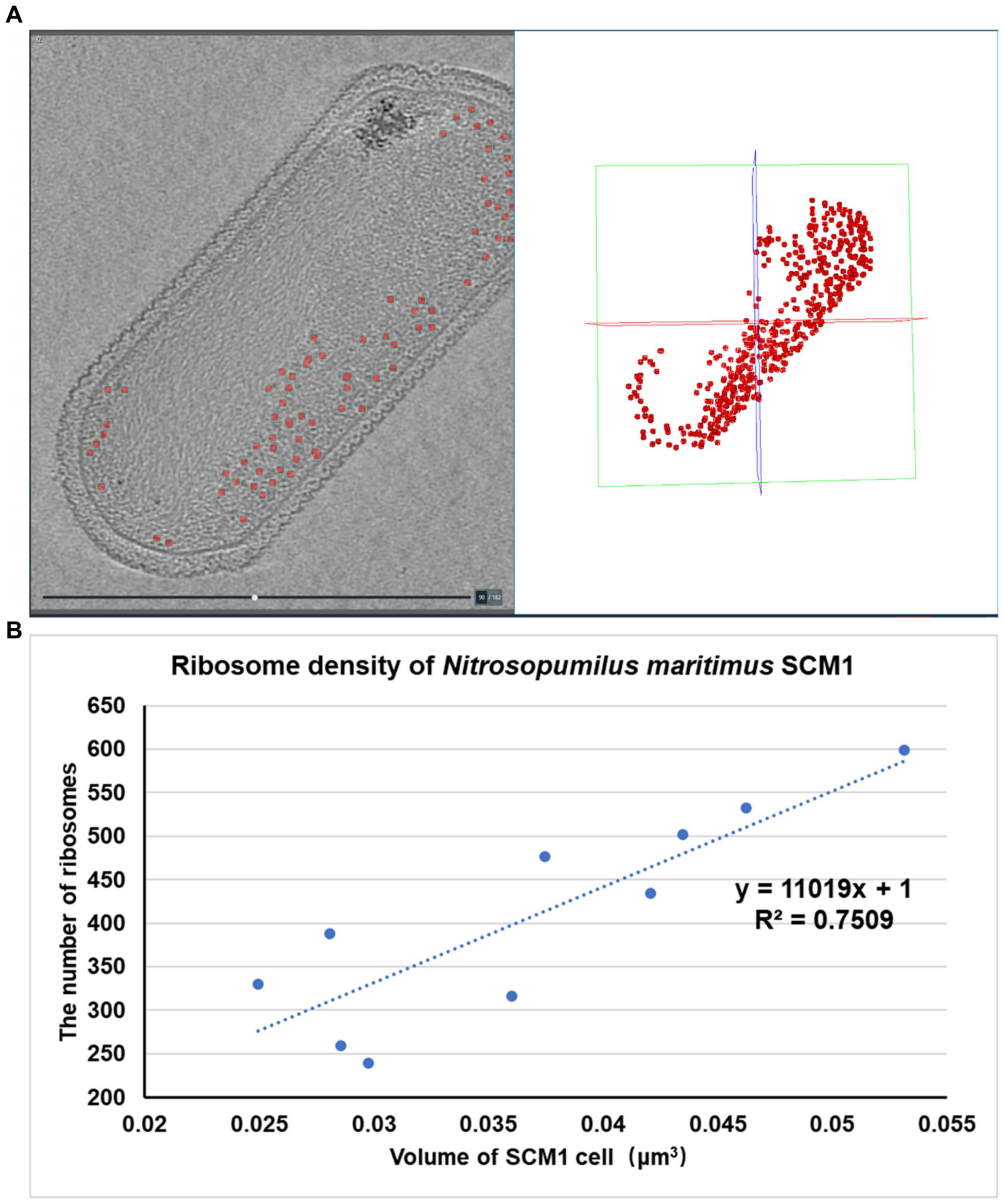
Spatial organization and density of ribosomes in SCM1 cells observed by cryo-ET. **(A)** Left: Central section through a tomogram showed the regions of ribosomes (indicated by red dots). Right: Regions of ribosomes observed by cryo-ET in the 3D volume. **(B)** The *x*-axis represents the volume of SCM1 cell (μm^3^), the *y*-axis represents the number of ribosomes in the cell. Trendline was plotted, the slope means the ribosome density of SCM1 cells.

The number of ribosomes observed in each SCM1 cell ranged between 200 and 600 (Figure 5), with an estimated ribosome diameter of 17–24 nm. These ribosomes were distributed around the cell membranes and located in the peripheral region of the nucleoid (Figures 1, 2). Notably, a linear relationship was identified between cell size and the number of ribosomes per cell (Figure 5). The number of ribosomes in SCM1 we observed was about 2–5 fold lower than what was reported by Urakawa et al. (2011).

In comparison to eukaryotes (Martiny et al., 2023) or other prokaryotes (Xue et al., 2022), SCM1 in this study contained significantly fewer ribosomes per cell. This observation supports the notion of the slow growth typically observed in laboratory culture (Supplementary Figure 1).

## Discussion

According to previous studies, SCM1 contains two cell division systems in the genome, but only uses one system, the Cdv system, correlated with the eukaryotic ESCRT-III machinery, for cell division (Pelve et al., 2011; van Wolferen et al., 2022). However, the function of FtsZ system in SCM1 even in the whole *Nitrososphaeria* phylum, is not yet fully understood. Only one study has reported that the absence of polymerization function prevents FtsZ from participating in the cell division process (Ng et al., 2013). Based on the cryo-EM images we captured, we not only observed SCM1 cells in the fission state but also identified cells potentially in the budding state (Figures 2E,F), suggesting the activation of the FtsZ system during the budding process (Liao et al., 2021). However, given the limited number of SCM1 cells we observed in the budding state, we cannot rule out the possibility of them being extracellular membrane vesicles, similar to those found in haloarchaea *Haloferax volcanii* (Deutschmann et al., 2001; Rock et al., 2017; Shalev et al., 2017).

Energy storage is crucial for microorganisms, providing resilience against fluctuations in nutrient availability. Our study utilized cryo-EM, cyro-ET, and STEM-EDS analysis to investigate storage granules in SCM1 cells, revealing distinct electron density and elemental compositions between the medium-density region (MDR) and high-density region (HDR) granules. Contrary to previous studies suggesting the absence of polyphosphate in SCM1 cells (Urakawa et al., 2011), our findings indicate its presence, suggesting a potential role in global phosphorus cycling (Qin et al., 2020). The initial introduction of EDS technique to biological samples enabled the analysis of similar storage granules in yeast and methanogenic archaea cells (Zierold, 1982; Scherer and Bochem, 1983). Subsequent studies have characterized high abundances of phosphorus, oxygen, and cationic elements in polyphosphate bodies in various archaeal species, including *Sulfolobus metallicus* (Remonsellez et al., 2006), *Methanospirillum hungatei* (Toso et al., 2011), *Archaeoglobus fulgidus* (Toso et al., 2016) and ANME-2b (McGlynn et al., 2018). Furthermore, the presence of magnesium and calcium in the HDR granules may facilitate the formation of ionic bonds with phosphate groups, contributing to the stability and density of these structures (Parsons et al., 2010). Additionally, the higher carbon content observed in the MDR granules may suggest their involvement in carbon storage or metabolism.

The presence of PHA in SCM1, as determined by STEM-EDS, Raman spectroscopy, genome and proteome analyses, opens avenues for understanding its physiological functions. While the exact function of PHA in SCM1 remains to be elucidated (Qin et al., 2020), its potential role in carbon and energy storage, similar to its function in haloarchaea, warrants further investigation. PHA in SCM1-like marine AOA may enhance their adaptation to periodic ammonia limitation in the oligotrophic environment. The 3-hydroxypropionate/4-hydroxybutyrate (3HP/4HB) cycle has only been discovered in archaea (Berg et al., 2007, 2010) and represents the most energy-efficient aerobic carbon fixation pathway currently known (Konneke et al., 2014). When carbon fixation is impeded due to a lack of energy sources, the degradation of PHA may sustain the basic metabolic requirements of marine AOA cells (Spang et al., 2012; Konneke et al., 2014; Abby et al., 2018). It would be interesting to conduct experiments to assess the expression levels of genes involved in PHA metabolic and carbon fixation pathways, and determine enzymatic activities under varying nutritional conditions. Further exploration of the role of PHA in SCM1’s metabolism promises valuable insights into its adaptive strategies.

The observed scarcity of ribosomes in *Nitrosopumilus maritimus* SCM1 is consistent with adaptation of SCM1 to the oligotrophic marine environment. Our results may prompt further investigation into the dynamic nature of ribosome abundance and its implications for the cellular physiology of this organism. Future research endeavors should focus on comprehensive ribosome characterization, shedding light on the regulatory mechanisms, which can be achieved by sub-tomogram averaging (Himes and Zhang, 2018; Ni et al., 2022). Studies on the native structures of ribosomes in bacterial and eukaryotic cells at high resolution (Erdmann et al., 2021; Hoffmann et al., 2022; Xue et al., 2022) may be applied to examine the fine structures of SCM1 ribosomes, particularly during the transcription and translation steps in protein synthesis (Xue et al., 2022) that limits SCM1 cell growth and division. Unraveling these intricacies would enhance our comprehension of how SCM1 thrives in the challenging conditions of the oligotrophic marine environment.

Cryo-ET is a pivotal technique for visualizing the 3D structure of cells or cellular organelles at nanoscale resolution. Traditionally, the specimen thickness suitable for cryo-ET is limited to ≤300 nm, a constraint that restricts its application to thinner biological samples (Varsano and Wolf, 2022). To address the limitations associated with specimen thickness and high tilt angles imaging, cryo-scanning transmission electron tomography (CSTET) has been introduced, extending the tomography study capacity to specimens up to 1 μm (Varsano and Wolf, 2022). Despite offering a broader application range for thicker specimens, CSTET comes with a trade-off, presenting a lower resolution compared to cryo-ET (Wolf and Elbaum, 2019). The quest for optimizing 3D imaging of vitrified specimens, however, continues. In this context, tilt-series cryo-ET remains a widely adopted method, primarily due to its proficiency in delivering high-resolution images of cellular ultrastructures (Wolf et al., 2014). In our study, we leverage a groundbreaking approach by employing spherical-chromatic-aberration-corrected cryo-ET. This innovative technique has significantly enhanced our ability to visualize cellular ultrastructures, enabling clear imaging of SCM1 cells with thicknesses extending up to 400 nm. This advancement not only showcases the potential for detailed observation of archaeal and bacterial cells but also illuminates the complex regions of eukaryotic cells that are ≤400 nm thick.

## Materials and methods

### *Nitrosopumilus maritimus* SCM1 cultivation

We followed previous protocol for SCM1 cultivation, growth monitoring, and culture purity assessment (Law et al., 2021b). To ensure the absence of bacterial contaminants during the long-term cultivation, we used a combination of 0.5 μg/mL Rifamycin sodium and 1 μg/mL hydrosoluble Amphotericin B. We assessed cell growth by detecting nitrite production, which was determined by diazo-colorimetric assay with photometric detection at 545 nm. For evaluating culture purity, we employed quantitative real-time PCR (qPCR) to accurately measure the culture’s integrity and absence of contaminants, detailed data are provided in Supplementary Table 1. The archaeal primer pairs for qPCR are ARC787F (ATTAGATACCCSBGTAGTCC) and ARC1059R (GCCATGCACCWCCTCT), while the bacterial primer pairs are BAC338F (ACTCCTACGGGAGGCAG) and BAC805R (GACTACCAGGGTATCTAATCC) (Yu et al., 2005).

### Collection and processing of Raman spectra of *Nitrosopumilus maritimus* SCM1

*Nitrosopumilus maritimus* SCM1 was incubated at 30°C for 14 days. The pre-treatment processes were as described previously (Wang et al., 2021). Single-cell Raman spectra were acquired using a Raman imaging microscope (WITec alpha 300R) with 532 nm laser. All raw spectra were preprocessed with Project Five 5.2 (WITec) for baseline correction and vector normalization.

### Cryo-EM sample preparation and data acquisition

Quantifoil Cu R2/1 grids were glow discharged at 15 mA for 45 s using a Pelco easiGlow discharged unit. An aliquot of 4 μL concentrated sample of SCM1 was applied to the front side of the grids, then the sample was vitrified by plunge freezing in liquid ethane using a Mark IV Vitrobot (Thermo Fisher Scientific) at 6°C and 100% humidity. The vitrified samples were loaded into a 300-kV Titan Krios microscopy G3i (Thermo Fisher Scientific) equipped with GIF quantum energy filter with a slit of 30 eV and K2 Summit direct electron detector (Gatan Inc.). Images were acquired using SerialEM package (Mastronarde, 2003) by the K2 camera operated under the super-resolution mode at a nominal magnification of ×26,000 (calibrated pixel size 5.417 Å). Each micrograph was fractionated into 36 frames with a total dose of 50 e^−^/Å^2^. The defocus range was set from −5.0 to −8.0 μm.

### Cryo-ET data collection and tomogram reconstruction

For cryo-ET, the vitrified samples were loaded into a 300-kV Titan Krios microscopy G4 (Thermo Fisher Scientific) equipped with Selectris energy filter with a slit of 50 eV and CEOS CCOR-spherical aberration/chromatic aberration (Cs/Cc) corrector. Tilt images were acquired by Falcon 4 camera under counting mode at a nominal magnification of ×26,000 (calibrated pixel size of 3.41 Å). A dose-symmetric scheme (Hagen et al., 2017) was used to collect tilt-series from −60° to +60° at a step size of 2° using SerialEM software (Mastronarde, 2003). Each tilt image was recorded as a video stack consisting of 10 frames with a dose of 1.64 e^−^/Å^2^. The defocus was set range from −4 to −6 μm and the total dose was 100 e^−^/Å^2^. Video frames of each tilt image were motion-corrected by MotionCor2 v.1.1.0 (Zheng et al., 2017). Tilt series were merged into one stack, aligned with patch-tracking in IMOD (v.4.9.12, Kremer et al., 1996) and reconstructed as back-projected tomograms with SIRT-like filtering of 5 iterations at bin4. In total, 221 sets of tilt-series were collected. The missing-wedge information was reconstructed using IsoNet in selected tomograms (Liu et al., 2022). The optimized tomograms were displayed and segmented by Amira (version 2020.3.1, Thermo Fisher Scientific). In the reconstructed tomograms, i.e., in the 3D volume of a given cell, ribosomes were identified and calculated manually based on their structural characteristics and electron density in Amira.

### STEM-EDS analysis

Samples were cryo-transferred by the Elsa™ holder (Gatan, model 698, ultra-low profile) and imaged in a Thermofisher Talos F200X transmission electron microscopy equipped with a Ceta camera in bright field mode, operated at a 200 kV acceleration voltage. Next, the cryo-holder with the sample was inserted into a pumping station to warm up to room temperature. Then a focused electron probe with high energy was used to stimulate a specific area of the sample. The atoms being stimulated may eject electrons, resulting in the emission of X-ray photons as a form of energy release. The energy exhibited by these X-rays is indicative of the particular atomic element from which they originated. Therefore, an energy-dispersive spectrometer can be employed to quantify the quantity and energy of the X-rays, providing a quantitative description of the elemental composition of the stimulated area of the sample (Toso et al., 2011). STEM-EDS images were acquired at room temperature. The data process and 3D visualization were performed by Inspect 3D and Avizo software.

### Gene and genomic analysis

Phylogenomic analysis of ammonia-oxidizing *Nitrososphaeria* was conducted using IQ-TREE (Minh et al., 2020) with GTDB archaeal markers obtained from the Genome Taxonomy Database (GTDB).^1^ The selected markers were carefully aligned using MAFFT (Katoh and Standley, 2013) and then concatenated into a supermatrix. The best-fitting substitution model was determined using ModelFinder (Kalyaanamoorthy et al., 2017), and the phylogenomic tree was constructed using Maximum Likelihood with ultrafast bootstrap approximation. The genomes were curated as our previous study (Pereira et al., 2024). The PolyP and PHA metabolic gene coded proteins were collected from archives (Spang et al., 2012; Wang et al., 2019; Mitra et al., 2020) and investigated in AOA after predicting proteins from the AOA genomes. The obtained AOA proteins were aligned against the KO profiles available in the KEGG database (accessed in November 2023). The results with the similarity higher than 90% were filtered based on the HMM score (Johnson et al., 2010), and the corresponding KO related to PolyP and PHA metabolic genes were searched. The KO numbers of PolyP related genes are: K00937(*Ppk1*), K22468(*Ppk2*), K23753(*Ppk2*), K01514(*PPX1*), K01524(*Ppx/GppA*), and PHA related genes are: K22881(*PhaE*), K03821(*PhaC*), K22250(*PhaB*), K24257(*PhaR*), K00019(*BdhA*), K19659(*PhaJ*), K03932(*PhaZ1*), K05973(*PhaZ*), K22249(*PhaZ*), K22250(*PhaZ*), K12405(hydratase), K17865(hydratase), K15016(hydratase), K00074(dehydrogenase), K00022(dehydrogenase). Additionally, to confirm our annotation, the presence of each key functional domain of the targeted proteins was manually checked using InterProScan.^2^ After the annotation and confirmation, the tree was enhanced using the Interactive Tree of Life (iTOL) web tool (Letunic and Bork, 2021). Branches were colorized, annotations were added, and the tree’s appearance was customized to improve clarity and aesthetics.

### Proteome analysis

Filters containing late exponential phase *Nitrosopumilus maritimus* SCM1 cells were extracted using a modified protocol as described before (Qin et al., 2018), either trypsin or GluC was used as protease digestion. Mass spectrometry analysis was carried out at Shenzhen Wininnovate BioTechnology Co., Ltd. DDA (data-dependent acquisition) mass spectrum techniques were used to acquire tandem MS data on a ThermoFisher Q Exactive mass spectrometer fitted with a Nano Flex ion source. The raw MS/MS spectra were treated and searched against the NCBI and UniProt Proteome dataset using MaxQuant 2.0.3.0. The generated data set contains the results of different hydrolases and databases for comparison.

## Data availability statement

The original contributions presented in the study are included in the article/Supplementary material, further inquiries can be directed to the corresponding authors.

## Author contributions

YaZ: Writing – review & editing, Writing – original draft, Validation, Methodology, Investigation, Formal analysis, Data curation. AY: Writing – review & editing, Writing – original draft, Validation, Methodology, Investigation, Formal analysis, Data curation. JY: Writing – review & editing, Writing – original draft, Visualization, Validation, Software, Methodology, Investigation, Formal analysis, Data curation. WH: Writing – review & editing, Writing – original draft, Visualization, Validation, Methodology, Investigation, Formal analysis, Data curation. SG: Writing – review & editing, Writing – original draft, Visualization, Validation, Methodology, Investigation, Formal analysis. YiL: Writing – review & editing, Validation, Investigation. JW: Writing – review & editing, Visualization, Validation, Investigation. YD: Writing – review & editing, Visualization, Validation, Investigation, Formal analysis, Data curation. XP: Writing – review & editing, Visualization, Validation, Resources, Investigation, Formal analysis. DC: Writing – review & editing, Validation, Investigation. OP: Investigation, Writing – review & editing, Validation. WT: Writing – review & editing, Validation, Investigation. RB: Writing – review & editing, Validation, Investigation. SC: Writing – review & editing, Validation, Investigation. LF: Writing – review & editing, Validation, Investigation. PW: Writing – review & editing, Validation, Resources, Project administration. YaL: Writing – review & editing, Validation, Formal analysis. WQ: Writing – review & editing, Validation, Formal analysis. S-FS: Writing – review & editing, Supervision, Resources, Project administration, Conceptualization. YuZ: Writing – review & editing, Validation, Supervision, Conceptualization. CZ: Writing – review & editing, Writing – original draft, Validation, Supervision, Resources, Project administration, Methodology, Funding acquisition, Conceptualization. ZL: Writing – review & editing, Writing – original draft, Validation, Supervision, Resources, Project administration, Methodology, Funding acquisition, Conceptualization.

## Footnotes

^1^http://gtdb.ecogenomic.org/

^2^https://www.ebi.ac.uk/interpro/

## References

[ref1] Abby S. S. Kerou M. Schleper C. (2020). Ancestral reconstructions decipher major adaptations of ammonia-oxidizing archaea upon radiation into moderate terrestrial and marine environments. MBio 11:e02371-20. doi: 10.1128/mBio.02371-20 33051370 PMC7554672

[ref2] Abby S. S. Melcher M. Kerou M. Krupovic M. Stieglmeier M. Rossel C.. (2018). *Candidatus* nitrosocaldus cavascurensis, an ammonia oxidizing, extremely thermophilic archaeon with a highly mobile genome. Front. Microbiol. 9:28. doi: 10.3389/fmicb.2018.00028, PMID: 29434576 PMC5797428

[ref3] Berg I. A. Kockelkorn D. Buckel W. Fuchs G. (2007). A 3-Hydroxypropionate/4-Hydroxybutyrate autotrophic carbon dioxide assimilation pathway in Archaea. Science 318, 1782–1786. doi: 10.1126/science.1149976, PMID: 18079405

[ref4] Berg I. A. Kockelkorn D. Ramos-Vera W. H. Say R. F. Zarzycki J. Hügler M.. (2010). Autotrophic carbon fixation in archaea. Nat. Rev. Microbiol. 8, 447–460. doi: 10.1038/nrmicro2365 20453874

[ref5] Brochier-Armanet C. Boussau B. Gribaldo S. Forterre P. (2008). Mesophilic crenarchaeota: proposal for a third archaeal phylum, the Thaumarchaeota. Nat. Rev. Microbiol. 6, 245–252. doi: 10.1038/nrmicro1852, PMID: 18274537

[ref6] Ciobotă V. Burkhardt E.-M. Schumacher W. Rösch P. Küsel K. Popp J. (2010). The influence of intracellular storage material on bacterial identification by means of Raman spectroscopy. Anal. Bioanal. Chem. 397, 2929–2937. doi: 10.1007/s00216-010-3895-1, PMID: 20582405

[ref7] Deutschmann I. M. Krabberød A. K. Latorre F. Delage E. Marrasé C. Balagué V.. (2001). Characterization of inverted membrane vesicles from the halophilic archaeon *Haloferax volcanii*. J. Membr. Biol. 183, 195–204. doi: 10.1007/s00232-001-0067-4, PMID: 11696861

[ref8] Erdmann P. S. Hou Z. Klumpe S. Khavnekar S. Beck F. Wilfling F.. (2021). In situ cryo-electron tomography reveals gradient organization of ribosome biogenesis in intact nucleoli. Nat. Commun. 12:5364. doi: 10.1038/s41467-021-25413-w, PMID: 34508074 PMC8433212

[ref9] Gal A. Sviben S. Wirth R. Schreiber A. Lassalle-Kaiser B. Faivre D.. (2017). Trace-element incorporation into intracellular pools uncovers calcium-pathways in a Coccolithophore. Adv. Sci. 4:1700088. doi: 10.1002/advs.201700088, PMID: 29051853 PMC5644232

[ref10] González-Pech R. A. Li V. Y. Garcia V. Boville E. Mammone M. Kitano H.. (2023). The evolution, assembly, and dynamics of marine Holobionts. Annu. Rev. Mar. Sci. 16, 443–466. doi: 10.1146/annurev-marine-022123-104345 37552896

[ref11] Hagen W. J. H. Wan W. Briggs J. A. G. (2017). Implementation of a cryo-electron tomography tilt-scheme optimized for high resolution subtomogram averaging. J. Struct. Biol. 197, 191–198. doi: 10.1016/j.jsb.2016.06.007, PMID: 27313000 PMC5287356

[ref12] Himes B. A. Zhang P. (2018). emClarity: software for high-resolution cryo-electron tomography and subtomogram averaging. Nat. Methods 15, 955–961. doi: 10.1038/s41592-018-0167-z, PMID: 30349041 PMC6281437

[ref13] Hodgskiss L. H. Melcher M. Kerou M. Chen W. Ponce-Toledo R. I. Savvides S. N.. (2023). Unexpected complexity of the ammonia monooxygenase in archaea. ISME J. 17, 588–599. doi: 10.1038/s41396-023-01367-3, PMID: 36721060 PMC10030591

[ref14] Hoffmann P. C. Kreysing J. P. Khusainov I. Tuijtel M. W. Welsch S. Beck M. (2022). Structures of the eukaryotic ribosome and its translational states in situ. Nat. Commun. 13:7435. doi: 10.1038/s41467-022-34997-w, PMID: 36460643 PMC9718845

[ref15] Johnson L. S. Eddy S. R. Portugaly E. (2010). Hidden Markov model speed heuristic and iterative HMM search procedure. BMC Bioinformatics 11:431. doi: 10.1186/1471-2105-11-431, PMID: 20718988 PMC2931519

[ref16] Kalyaanamoorthy S. Minh B. Q. Wong T. K. F. Von Haeseler A. Jermiin L. S. (2017). ModelFinder: fast model selection for accurate phylogenetic estimates. Nat. Methods 14, 587–589. doi: 10.1038/nmeth.4285, PMID: 28481363 PMC5453245

[ref17] Katoh K. Standley D. M. (2013). MAFFT multiple sequence alignment software version 7: improvements in performance and usability. Mol. Biol. Evol. 30, 772–780. doi: 10.1093/molbev/mst010, PMID: 23329690 PMC3603318

[ref18] Kim J.-G. Kim S.-J. Cvirkaite-Krupovic V. Yu W.-J. Gwak J.-H. López-Pérez M.. (2019). Spindle-shaped viruses infect marine ammonia-oxidizing thaumarchaea. Proc. Natl. Acad. Sci. U. S. A. 116, 15645–15650. doi: 10.1073/pnas.1905682116, PMID: 31311861 PMC6681747

[ref19] Kitzinger K. Marchant H. K. Bristow L. A. Herbold C. W. Padilla C. C. Kidane A. T.. (2020). Single cell analyses reveal contrasting life strategies of the two main nitrifiers in the ocean. Nat. Commun. 11:767. doi: 10.1038/s41467-020-14542-3, PMID: 32034151 PMC7005884

[ref20] Kitzinger K. Padilla C. C. Marchant H. K. Hach P. F. Herbold C. W. Kidane A. T.. (2019). Cyanate and urea are substrates for nitrification by Thaumarchaeota in the marine environment. Annu. Rev. Earth Planet. Sci. 4, 234–243. doi: 10.1038/s41564-018-0316-2, PMID: 30531977 PMC6825518

[ref21] Konneke M. Bernhard A. E. de la Torre J. R. Walker C. B. Waterbury J. B. Stahl D. A. (2005). Isolation of an autotrophic ammonia-oxidizing marine archaeon. Nature 437, 543–546. doi: 10.1038/nature03911, PMID: 16177789

[ref22] Konneke M. Schubert D. M. Brown P. C. Hugler M. Standfest S. Schwander T.. (2014). Ammonia-oxidizing archaea use the most energy-efficient aerobic pathway for CO_2_ fixation. Proc. Natl. Acad. Sci. U. S. A. 111, 8239–8244. doi: 10.1073/pnas.1402028111, PMID: 24843170 PMC4050595

[ref23] Kraft B. Jehmlich N. Larsen M. Bristow L. A. Könneke M. Thamdrup B.. (2022). Oxygen and nitrogen production by an ammonia-oxidizing archaeon. Science 375, 97–100. doi: 10.1126/science.abe6733, PMID: 34990242

[ref24] Kremer J. R. Mastronarde D. N. McIntosh J. R. (1996). Computer visualization of three-dimensional image data using IMOD. J. Struct. Biol. 116, 71–76. doi: 10.1006/jsbi.1996.0013, PMID: 8742726

[ref25] Law K. P. He W. Tao J. Zhang C. (2021a). A novel approach to characterize the Lipidome of marine archaeon *Nitrosopumilus maritimus* by ion mobility mass spectrometry. Front. Microbiol. 12:735878. doi: 10.3389/fmicb.2021.735878 34925256 PMC8674956

[ref26] Law K. P. He W. Tao J. Zhang C. (2021b). Characterization of the exometabolome of *Nitrosopumilus maritimus* SCM1 by liquid chromatography–ion mobility mass spectrometry. Front. Microbiol. 12:658781. doi: 10.3389/fmicb.2021.658781 34276593 PMC8281238

[ref27] Leavitt W. D. Kopf S. H. Weber Y. Chiu B. McFarlin J. M. Elling F. J.. (2023). Controls on the hydrogen isotope composition of tetraether lipids in an autotrophic ammonia-oxidizing marine archaeon. Geochim. Cosmochim. Acta 352, 194–210. doi: 10.1016/j.gca.2023.04.033

[ref28] Letunic I. Bork P. (2021). Interactive tree of life (iTOL) v5: an online tool for phylogenetic tree display and annotation. Nucleic Acids Res. 49, W293–W296. doi: 10.1093/nar/gkab301, PMID: 33885785 PMC8265157

[ref29] Li P.-N. Herrmann J. Tolar B. B. Poitevin F. Ramdasi R. Bargar J. R.. (2018). Nutrient transport suggests an evolutionary basis for charged archaeal surface layer proteins. ISME J. 12, 2389–2402. doi: 10.1038/s41396-018-0191-0, PMID: 29899515 PMC6155111

[ref30] Liao Y. Ithurbide S. Evenhuis C. Löwe J. Duggin I. G. (2021). Cell division in the archaeon *Haloferax volcanii* relies on two FtsZ proteins with distinct functions in division ring assembly and constriction. Nat. Microbiol. 6, 594–605. doi: 10.1038/s41564-021-00894-z, PMID: 33903747 PMC7611241

[ref31] Lipp J. S. Morono Y. Inagaki F. Hinrichs K.-U. (2008). Significant contribution of Archaea to extant biomass in marine subsurface sediments. Nature 454, 991–994. doi: 10.1038/nature07174, PMID: 18641632

[ref32] Liu Y.-T. Zhang H. Wang H. Tao C.-L. Bi G.-Q. Zhou Z. H. (2022). Isotropic reconstruction for electron tomography with deep learning. Nat. Commun. 13:6482. doi: 10.1038/s41467-022-33957-8, PMID: 36309499 PMC9617606

[ref33] Martens-Habbena W. Berube P. M. Urakawa H. de la Torre J. R. Stahl D. A. (2009). Ammonia oxidation kinetics determine niche separation of nitrifying Archaea and Bacteria. Nature 461, 976–979. doi: 10.1038/nature08465, PMID: 19794413

[ref34] Martikainen P. J. (2022). Heterotrophic nitrification – an eternal mystery in the nitrogen cycle. Soil Biol. Biochem. 168:108611. doi: 10.1016/j.soilbio.2022.108611

[ref35] Martiny J. B. H. Martiny A. C. Brodie E. Chase A. B. Rodríguez-Verdugo A. Treseder K. K.. (2023). Translation dynamics in human cells visualized at high resolution reveal cancer drug action. Science 381, 70–75. doi: 10.1126/science.adh1411, PMID: 37410833

[ref36] Mastronarde D. N. (2003). SerialEM: a program for automated tilt series acquisition on Tecnai microscopes using prediction of specimen position. Microsc. Microanal. 9, 1182–1183. doi: 10.1017/s1431927603445911

[ref37] McGlynn S. E. Chadwick G. L. O’Neill A. Mackey M. Thor A. Deerinck T. J.. (2018). Subgroup characteristics of marine methane-oxidizing ANME-2 Archaea and their syntrophic partners as revealed by integrated multimodal analytical microscopy. Appl. Environ. Microbiol. 84:e00399-18. doi: 10.1128/AEM.00399-18, PMID: 29625978 PMC5960974

[ref38] Minh B. Q. Schmidt H. A. Chernomor O. Schrempf D. Woodhams M. D. von Haeseler A.. (2020). IQ-TREE 2: new models and efficient methods for phylogenetic inference in the genomic era. Mol. Biol. Evol. 37, 1530–1534. doi: 10.1093/molbev/msaa015, PMID: 32011700 PMC7182206

[ref39] Mitra R. Xu T. Xiang H. Han J. (2020). Current developments on polyhydroxyalkanoates synthesis by using halophiles as a promising cell factory. Microb. Cell Factories 19, 86–30. doi: 10.1186/s12934-020-01342-z, PMID: 32264891 PMC7137286

[ref40] Ng K.-H. Srinivas V. Srinivasan R. Balasubramanian M. (2013). The *Nitrosopumilus maritimus* CdvB, but not FtsZ, assembles into polymers. Archaea 2013, 1–10. doi: 10.1155/2013/104147, PMID: 23818813 PMC3684127

[ref41] Ni T. Frosio T. Mendonça L. Sheng Y. Clare D. Himes B. A.. (2022). High-resolution in situ structure determination by cryo-electron tomography and subtomogram averaging using emClarity. Nat. Protoc. 17, 421–444. doi: 10.1038/s41596-021-00648-5, PMID: 35022621 PMC9251519

[ref42] Oren A. Garrity G. M. (2021). Valid publication of the names of forty-two phyla of prokaryotes. Int. J. Syst. Evol. Microbiol. 71:5056. doi: 10.1099/ijsem.0.005056, PMID: 34694987

[ref43] Parsons A. J. Ahmed I. Rudd C. D. Cuello G. J. Pellegrini E. Richard D.. (2010). Neutron scattering and*ab initio*molecular dynamics study of cross-linking in biomedical phosphate glasses. J. Phys. Condens. Matter 22:485403. doi: 10.1088/0953-8984/22/48/485403, PMID: 21406745

[ref44] Pelve E. A. Lindås A.-C. Martens-Habbena W. de la Torre J. R. Stahl D. A. Bernander R. (2011). Cdv-based cell division and cell cycle organization in the thaumarchaeon *Nitrosopumilus maritimus*. Mol. Microbiol. 82, 555–566. doi: 10.1111/j.1365-2958.2011.07834.x, PMID: 21923770

[ref45] Pereira O. Qin W. Galand P. E. Debroas D. Lami R. Hochart C.. (2024). Metabolic activities of marine ammonia-oxidizing archaea orchestrated by quorum sensing. Review. [Preprint] doi: 10.21203/rs.3.rs-3718467/v2 PMC11442133 39359677

[ref46] Qin W. Amin S. A. Lundeen R. A. Heal K. R. Martens-Habbena W. Turkarslan S.. (2018). Stress response of a marine ammonia-oxidizing archaeon informs physiological status of environmental populations. ISME J. 12, 508–519. doi: 10.1038/ismej.2017.186, PMID: 29053148 PMC5776466

[ref47] Qin W. Amin S. A. Martens-Habbena W. Walker C. B. Urakawa H. Devol A. H.. (2014). Marine ammonia-oxidizing archaeal isolates display obligate mixotrophy and wide ecotypic variation. Proc. Natl. Acad. Sci. U. S. A. 111, 12504–12509. doi: 10.1073/pnas.1324115111, PMID: 25114236 PMC4151751

[ref48] Qin W. Heal K. R. Ramdasi R. Kobelt J. N. Martens-Habbena W. Bertagnolli A. D.. (2017). *Nitrosopumilus maritimus* gen. nov., sp. nov., *Nitrosopumilus cobalaminigenes* sp. nov., *Nitrosopumilus oxyclinae* sp. nov., and *Nitrosopumilus ureiphilus* sp. nov., four marine ammonia-oxidizing archaea of the phylum Thaumarchaeota. Int. J. Syst. Evol. Microbiol. 67, 5067–5079. doi: 10.1099/ijsem.0.002416, PMID: 29034851

[ref49] Qin W. Zheng Y. Zhao F. Wang Y. Urakawa H. Martens-Habbena W.. (2020). Alternative strategies of nutrient acquisition and energy conservation map to the biogeography of marine ammonia-oxidizing archaea. ISME J. 14, 2595–2609. doi: 10.1038/s41396-020-0710-7, PMID: 32636492 PMC7490402

[ref50] Remonsellez F. Orell A. Jerez C. A. (2006). Copper tolerance of the thermoacidophilic archaeon *Sulfolobus metallicus*: possible role of polyphosphate metabolism. Microbiology 152, 59–66. doi: 10.1099/mic.0.28241-0, PMID: 16385115

[ref51] Rinke C. Chuvochina M. Mussig A. J. Chaumeil P.-A. Davín A. A. Waite D. W.. (2021). A standardized archaeal taxonomy for the genome taxonomy database. Nat. Microbiol. 6, 946–959. doi: 10.1038/s41564-021-00918-8, PMID: 34155373

[ref52] Rock R. R. Turnbaugh P. J. Satinsky B. M. Smith C. B. Sharma S. Ward N. D.. (2017). N-glycosylation is important for proper *Haloferax volcanii* S-layer stability and function. Appl. Environ. Microbiol. 83:e03152-16. doi: 10.1128/AEM.03152-16, PMID: 28039139 PMC5335521

[ref53] Sarkar P. Shukla M. R. Kumbhar P. Manjre S. Dasgupta S. Bhakthavatsalam V. (2021). “Intracellular localization of micronutrients in algae cells using scanning transmission electron microscopy–energy-dispersive X-ray spectroscopy (STEM-EDX)” in Applications of microscopy in materials and life sciences. eds. Ghosal P. Carter C. B. Vinothkumar K. R. Sarkar R. (Singapore: Springer), 203–210.

[ref54] Scherer P. A. Bochem H.-P. (1983). Ultrastructural investigation of 12 *Methanosarcinae* and related species grown on methanol for occurrence of polyphosphatelike inclusions. Can. J. Microbiol. 29, 1190–1199. doi: 10.1139/m83-182

[ref55] Shalev Y. Turgeman-Grott I. Tamir A. Eichler J. Gophna U. (2017). Cell surface glycosylation is required for efficient mating of *Haloferax volcanii*. Front. Microbiol. 8:1253. doi: 10.3389/fmicb.2017.01253, PMID: 28725221 PMC5496957

[ref56] Shin D. S. Pratt A. J. Tainer J. A. (2014). Archaeal genome guardians give insights into eukaryotic DNA replication and damage response proteins. Archaea 2014, 1–24. doi: 10.1155/2014/206735, PMID: 24701133 PMC3950489

[ref57] Spang A. Poehlein A. Offre P. Zumbrägel S. Haider S. Rychlik N.. (2012). The genome of the ammonia-oxidizing *Candidatus* Nitrososphaera gargensis: insights into metabolic versatility and environmental adaptations. Environ. Microbiol. 14, 3122–3145. doi: 10.1111/j.1462-2920.2012.02893.x, PMID: 23057602

[ref58] Stahl D. A. de la Torre J. R. (2012). Physiology and diversity of Ammonia-oxidizing Archaea. Ann. Rev. Microbiol. 66, 83–101. doi: 10.1146/annurev-micro-092611-150128 22994489

[ref59] Stieglmeier M. Klingl A. Alves R. J. E. Rittmann S. K.-M. R. Melcher M. Leisch N.. (2014). *Nitrososphaera viennensis* gen. nov., sp. nov., an aerobic and mesophilic, ammonia-oxidizing archaeon from soil and a member of the archaeal phylum Thaumarchaeota. Int. J. Syst. Evol. Microbiol. 64, 2738–2752. doi: 10.1099/ijs.0.063172-0, PMID: 24907263 PMC4129164

[ref60] Tocheva E. I. Dekas A. E. McGlynn S. E. Morris D. Orphan V. J. Jensen G. J. (2013). Polyphosphate storage during sporulation in the gram-negative bacterium *Acetonema longum*. J. Bacteriol. 195, 3940–3946. doi: 10.1128/JB.00712-13, PMID: 23813732 PMC3754598

[ref61] Toso D. B. Henstra A. M. Gunsalus R. P. Zhou Z. H. (2011). Structural, mass and elemental analyses of storage granules in methanogenic archaeal cells. Environ. Microbiol. 13, 2587–2599. doi: 10.1111/j.1462-2920.2011.02531.x, PMID: 21854518 PMC3700383

[ref62] Toso D. B. Javed M. M. Czornyj E. Gunsalus R. P. Zhou Z. H. (2016). Discovery and characterization of Iron sulfide and polyphosphate bodies coexisting in *Archaeoglobus fulgidus* cells. Archaea 2016, 1–11. doi: 10.1155/2016/4706532, PMID: 27194953 PMC4853940

[ref63] Treusch A. H. Leininger S. Kletzin A. Schuster S. C. Klenk H. Schleper C. (2005). Novel genes for nitrite reductase and Amo-related proteins indicate a role of uncultivated mesophilic crenarchaeota in nitrogen cycling. Environ. Microbiol. 7, 1985–1995. doi: 10.1111/j.1462-2920.2005.00906.x, PMID: 16309395

[ref64] Urakawa H. Martens-Habbena W. Stahl D. A. (2011). “Physiology and genomics of ammonia-oxidizing archaea” in Nitrification. eds. Ward B. B. Arp D. J. Klotz M. G. (Washington, D.C., United States: ASM Press), 115–155.

[ref65] van Wolferen M. Pulschen A. A. Baum B. Gribaldo S. Albers S.-V. (2022). The cell biology of archaea. Nat. Microbiol. 7, 1744–1755. doi: 10.1038/s41564-022-01215-8, PMID: 36253512 PMC7613921

[ref66] Varsano N. Wolf S. G. (2022). Electron microscopy of cellular ultrastructure in three dimensions. Curr. Opin. Struct. Biol. 76:102444. doi: 10.1016/j.sbi.2022.102444, PMID: 36041268

[ref67] Walker C. B. de la Torre J. R. Klotz M. G. Urakawa H. Pinel N. Arp D. J.. (2010). *Nitrosopumilus maritimus* genome reveals unique mechanisms for nitrification and autotrophy in globally distributed marine crenarchaea. Proc. Natl. Acad. Sci. U. S. A. 107, 8818–8823. doi: 10.1073/pnas.0913533107 20421470 PMC2889351

[ref68] Wan X. S. Hou L. Kao S.-J. Zhang Y. Sheng H.-X. Shen H.. (2023). Pathways of N_2_O production by marine ammonia-oxidizing archaea determined from dual-isotope labeling. Proc. Natl. Acad. Sci. U. S. A. 120:e2220697120. doi: 10.1073/pnas.2220697120, PMID: 36888658 PMC10243131

[ref69] Wang L. Liu Q. Wu X. Huang Y. Wise M. J. Liu Z.. (2019). Bioinformatics analysis of metabolism pathways of archaeal energy reserves. Sci. Rep. 9:1034. doi: 10.1038/s41598-018-37768-0, PMID: 30705313 PMC6355812

[ref70] Wang Y. Xu J. Cui D. Kong L. Chen S. Xie W.. (2021). Classification and identification of archaea using single-cell Raman ejection and artificial intelligence: implications for investigating uncultivated microorganisms. Anal. Chem. 93, 17012–17019. doi: 10.1021/acs.analchem.1c03495, PMID: 34910467

[ref71] Ward S. K. Heintz J. A. Albrecht R. M. Talaat A. M. (2012). Single-cell elemental analysis of Bacteria: quantitative analysis of polyphosphates in *Mycobacterium tuberculosis*. Front. Cell. Inf. Microbiol. 2:63. doi: 10.3389/fcimb.2012.00063, PMID: 22919654 PMC3417655

[ref72] Wolf S. G. Elbaum M. (2019). “Chapter 10 – CryoSTEM tomography in biology” in Methods in cell biology. eds. Müller-Reichert T. Pigino G. (Cambridge, Massachusetts, United States: Academic Press), 197–215. 10.1016/bs.mcb.2019.04.001 31326021

[ref73] Wolf S. G. Houben L. Elbaum M. (2014). Cryo-scanning transmission electron tomography of vitrified cells. Nat. Methods 11, 423–428. doi: 10.1038/nmeth.2842, PMID: 24531421

[ref74] Xue L. Lenz S. Zimmermann-Kogadeeva M. Tegunov D. Cramer P. Bork P.. (2022). Visualizing translation dynamics at atomic detail inside a bacterial cell. Nature 610, 205–211. doi: 10.1038/s41586-022-05255-2, PMID: 36171285 PMC9534751

[ref75] Yang Y. Zhang C. Lenton T. M. Yan X. Zhu M. Zhou M.. (2021). The evolution pathway of ammonia-oxidizing archaea shaped by major geological events. Mol. Biol. Evol. 38, 3637–3648. doi: 10.1093/molbev/msab129, PMID: 33993308 PMC8382903

[ref76] Yin Z. Bi X. Xu C. (2018). Ammonia-oxidizing Archaea (AOA) play with Ammonia-oxidizing Bacteria (AOB) in nitrogen removal from wastewater. Archaea 2018, 1–9. doi: 10.1155/2018/8429145, PMID: 30302054 PMC6158934

[ref77] Yu Y. Lee C. Kim J. Hwang S. (2005). Group-specific primer and probe sets to detect methanogenic communities using quantitative real-time polymerase chain reaction. Biotechnol. Bioeng. 89, 670–679. doi: 10.1002/bit.20347, PMID: 15696537

[ref78] Zheng S. Q. Palovcak E. Armache J.-P. Verba K. A. Cheng Y. Agard D. A. (2017). MotionCor2: anisotropic correction of beam-induced motion for improved cryo-electron microscopy. Nat. Methods 14, 331–332. doi: 10.1038/nmeth.4193, PMID: 28250466 PMC5494038

[ref79] Zierold K. (1982). Preparation of biological cryosections for analytical electron microscopy. Ultramicroscopy 10, 45–53. doi: 10.1016/0304-3991(82)90186-3, PMID: 7135624

