# Supplementary material for: Ultrastructural insights into cellular organization, energy storage and ribosomal dynamics of an ammonia-oxidizing archaeon from oligotrophic oceans
Source: Front Microbiol. 2024 Apr 26;15:1367658. doi: 10.3389/fmicb.2024.1367658 (PMC11082331; doi:10.3389/fmicb.2024.1367658)
Supplement: Supplementary file 1 [file Data_Sheet_1.pdf]

Supplementary Information

Supplementary Figure 1. Growth curve of *Nitrosopumilus maritimus* SCM1 with 1mM of primary NH_4 concentration.

Supplementary Figure 2. Statistically generated chart depicting the size range of SCM1 cells.

Supplementary Figure 3. Images of *Nitrosopumilus maritimus* SCM1 with or without spherical-chromatic-aberration-corrector on a Titan Krios G4 microscope equipped with a Falcon 4 direct electron detector.

Supplementary Table 1. The purity of the SCM1 cultures was evaluated using the qPCR.

Supplementary Table 2. The STEM-EDS data of selected *Nitrosopumilus maritimus* SCM1 cells.

Supplementary Table 3. The proteome data of *Nitrosopumilus maritimus* SCM1.

Supplementary Movie 1. Reconstructed 3D cryo-tomogram of a typical cell of *Nitrosopumilus maritimus* SCM1.

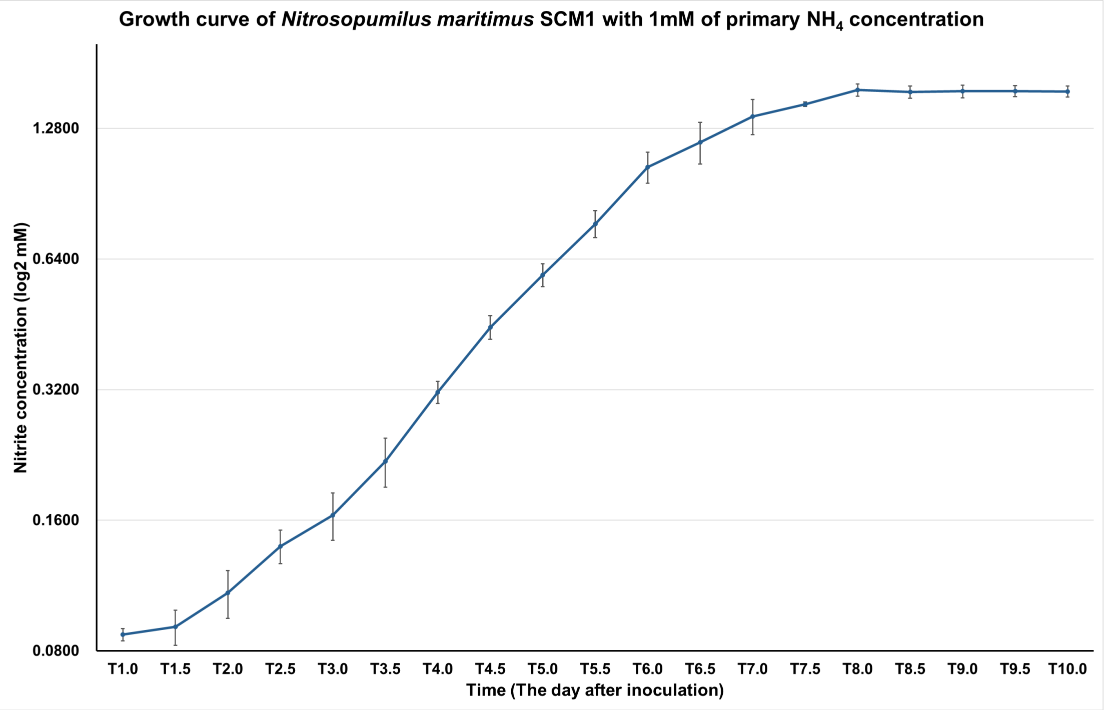

Supplementary Figure 1. Growth curve of *Nitrosopumilus maritimus* SCM1 with 1mM of primary NH_4 concentration.

We monitored cell growth in *Nitrosopumilus maritimus* SCM1 cultures by analyzing nitrite concentration. Nitrite concentration was continued recorded for every 12 hours after inoculation. The growth curve was plotted from 6 replicates with means ± SDs (error bars).

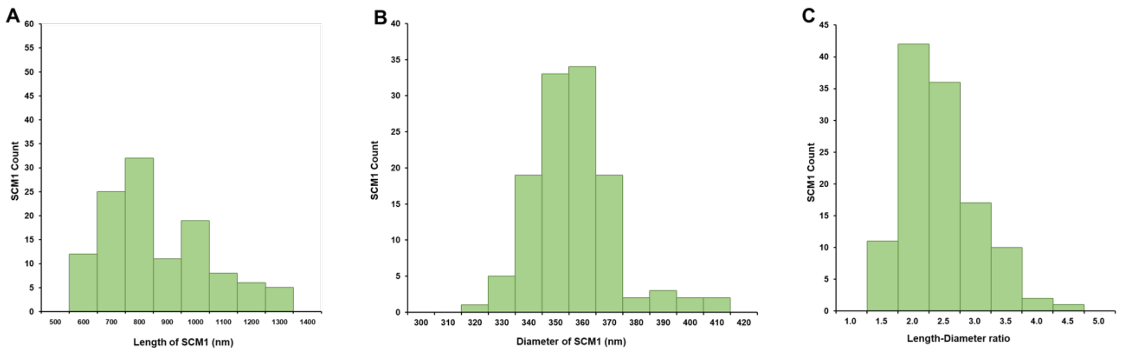

Supplementary Figure 2. Statistically generated chart depicting the size range of SCM1 cells.

The y-axis represents the number of SCM1 cells and the x-axis in each panel represents (A) Length (nm), (B) Diameter (nm), (C) Lenth-Diameter ratio of SCM1. The average length was 860 ± 181 nm (maximum: 1349 nm, minimum: 593 nm). The average diameter was 357 ± 15 nm (maximum: 412 nm, minimum 324 nm). The mean length-diameter ratio was 2.43 ± 0.57.

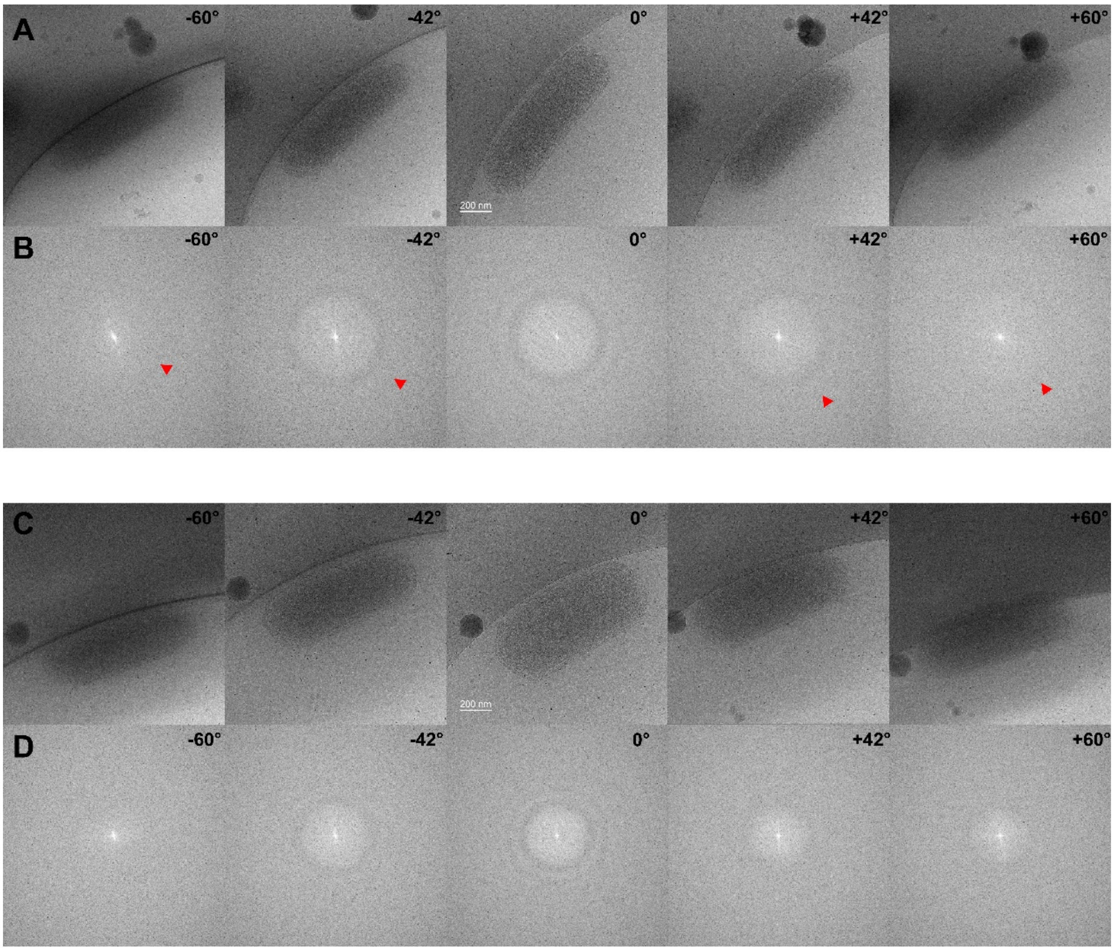

Supplementary Figure 3. Images of *Nitrosopumilus maritimus* SCM1 with or without spherical-chromatic-aberration-corrector on a Titan Krios G4 microscope equipped with a Falcon 4 direct electron detector.

(A) Cryo-EM images of SCM1 at -60°, -42°, 0°, +42°, and +60° angles with CCOR. (B) FFT of the above micrographs. Red arrowheads indicate extra Thon rings. (C) Cryo-EM images of SCM1 at -60°, -42°, 0°, +42°, and +60° angles without CCOR. (D) FFT of the above micrographs.

Supplementary Table 1. The purity of the SCM1 cultures was evaluated using the qPCR.

Sample	Mean copies of Bacteria	Mean copies of Archaea	Percentage of Contaminating Bacteria (%)
SCM1-1-220716	3,320.15	101,518,853.33	<0.001
SCM1-1-220801	2,822.03	127,649,797.33	<0.001
SCM1-1-220915	4,408.67	225,270,208.00	<0.001
SCM1-1-221110	3,348.08	136,850,829.33	<0.001
SCM1-1-221124	2225.04	126,674,933.33	<0.001
SCM1-1-221219	591.78	240,940,053.33	<0.001
SCM1-1-230212	746.39	63,871,446.00	<0.001
SCM1-1-230424	12,914.45	719,556,885.33	<0.001
SCM1-1-230515	9,383.56	212,321,997.33	<0.001
SCM1-1-231009	1,350.29	101,788,965.33	<0.001

Supplementary Table 2. The STEM-EDS data of selected *Nitrosopumilus maritimus* SCM1 cells.

In Figure 3A, two areas were emphasized:

Area 1	Z	Element	Family	Atomic Fraction (%)	Atomic Error (%)	Mass Fraction (%)	Mass Error (%)	Fit Error (%)
	6	C	K	56.37	2.76	44.71	2.4	3.1
	7	N	K	11.62	2.12	10.75	1.99	1.12
	8	O	K	19.74	3.23	20.86	3.37	0.62
	11	Na	K	3.57	0.71	5.43	1.07	1.98
	12	Mg	K	2.02	0.41	3.24	0.65	2.59
	13	Al	K	0.12	0.04	0.22	0.07	24.03
	14	Si	K	0.19	0.05	0.35	0.09	17.18
	15	P	K	3.27	0.63	6.7	1.24	1.36
	16	S	K	0.48	0.1	1.01	0.21	7.27
	17	Cl	K	0.75	0.15	1.75	0.34	5.2
	20	Ca	K	1.81	0.26	4.8	0.66	1.69
	26	Fe	K	0.05	0.02	0.19	0.07	33.87

Area 2	Z	Element	Family	Atomic Fraction (%)	Atomic Error (%)	Mass Fraction (%)	Mass Error (%)	Fit Error (%)
	6	C	K	67.53	2.53	58.89	2.48	1.42
	7	N	K	14.51	2.51	14.75	2.56	0.3
	8	O	K	10.52	1.92	12.22	2.19	1.37
	11	Na	K	4.68	0.91	7.82	1.47	0.25
	12	Mg	K	0.59	0.12	1.04	0.21	0.54
	13	Al	K	0.04	0.01	0.07	0.02	3.42
	14	Si	K	0.07	0.01	0.15	0.03	2.7
	15	P	K	0.97	0.19	2.17	0.41	0.31
	16	S	K	0.32	0.06	0.74	0.14	0.39
	17	Cl	K	0.48	0.09	1.24	0.23	0.36
	20	Ca	K	0.25	0.03	0.72	0.1	0.53
	26	Fe	K	0.05	0.01	0.18	0.03	1.39

In Figure 3B, six areas were emphasized:

Area 2	Z	Element	Family	Atomic Fraction (%)	Atomic Error (%)	Mass Fraction (%)	Mass Error (%)	Fit Error (%)
	6	C	K	73.37	2.77	63.59	3.12	10.75
	7	N	K	7.07	1.48	7.15	1.47	5.43
	8	O	K	7.93	1.6	9.16	1.8	2
	9	F	K	4.69	1	6.43	1.33	5.35
	11	Na	K	2.56	0.55	4.25	0.88	3.41
	12	Mg	K	1.25	0.27	2.19	0.47	4.74
	13	Al	K	0.2	0.07	0.4	0.13	24.92
	14	Si	K	1.46	0.31	2.97	0.61	4.61
	15	P	K	0.27	0.08	0.61	0.17	18.36
	16	S	K	0.36	0.09	0.84	0.2	13
	17	Cl	K	0.32	0.08	0.81	0.2	14.21
	19	K	K	0.11	0.04	0.31	0.13	35.4
	20	Ca	K	0.26	0.05	0.74	0.14	11.38
	26	Fe	K	0.14	0.04	0.55	0.14	21.58

Area 3	Z	Element	Family	Atomic Fraction (%)	Atomic Error (%)	Mass Fraction (%)	Mass Error (%)	Fit Error (%)
	6	C	K	67.85	5.21	57.43	5.75	21.65
	7	N	K	8.01	1.99	7.9	1.87	7.99
	8	O	K	11.05	2.61	12.46	2.76	5.04
	9	F	K	5.32	1.42	7.12	1.8	12.07
	11	Na	K	2.96	0.79	4.8	1.21	10.79
	12	Mg	K	1.04	0.34	1.79	0.56	20.98
	13	Al	K	0.35	0.21	0.67	0.4	55.49
	14	Si	K	1.26	0.37	2.5	0.7	16.93
	15	P	K	0.55	0.23	1.21	0.49	33.37
	16	S	K	0.45	0.2	1.03	0.45	37.49
	17	Cl	K	0.66	0.23	1.65	0.54	24.84
	19	K	K	0.11	0.16	0.3	0.43	141.63
	20	Ca	K	0.3	0.13	0.86	0.36	37.37
	26	Fe	K	0.07	0.12	0.28	0.46	162.32

Area 4	Z	Element	Family	Atomic Fraction (%)	Atomic Error (%)	Mass Fraction (%)	Mass Error (%)	Fit Error (%)
	6	C	K	71.77	3.1	61.61	3.45	11.29
	7	N	K	5.83	1.28	5.84	1.26	7.77
	8	O	K	11.71	2.31	13.39	2.55	3.27
	9	F	K	3.7	0.92	5.03	1.21	13.45
	11	Na	K	2.19	0.52	3.61	0.83	10.24
	12	Mg	K	0.9	0.25	1.57	0.43	17.68
	13	Al	K	0.27	0.15	0.52	0.28	50.02

	14	Si	K	1.78	0.4	3.58	0.78	8.69
	15	P	K	0.31	0.14	0.68	0.31	40.95
	16	S	K	0.34	0.14	0.79	0.32	35.65
	17	Cl	K	0.74	0.19	1.88	0.48	16.65
	19	K	K	0.12	0.11	0.32	0.31	93.69
	20	Ca	K	0.12	0.08	0.35	0.23	64.52
	26	Fe	K	0.21	0.09	0.84	0.35	39.33

**Area 5**	**Z**	**Element**	**Family**	**Atomic Fraction (%)**	**Atomic Error (%)**	**Mass Fraction (%)**	**Mass Error (%)**	**Fit Error (%)**
	6	C	K	35.45	5.65	24.51	4.5	22.88
	7	N	K	7.55	1.69	6.09	1.34	8.58
	8	O	K	23.22	4.15	21.38	3.72	2.67
	9	F	K	12.44	2.58	13.61	2.68	6.1
	11	Na	K	7.89	1.69	10.45	2.1	4.6
	12	Mg	K	3.94	0.9	5.51	1.2	7.41
	13	Al	K	0.16	0.2	0.25	0.31	126.6
	14	Si	K	1.26	0.34	2.04	0.54	16.84
	15	P	K	4.05	0.87	7.22	1.46	5.78
	16	S	K	0.65	0.23	1.2	0.41	27.97
	17	Cl	K	0.7	0.23	1.42	0.45	24.96
	19	K	K	0.04	0.16	0.1	0.35	350.46
	20	Ca	K	2.54	0.44	5.85	0.92	5.2
	26	Fe	K	0.12	0.12	0.37	0.39	103.72

**Area 6**	**Z**	**Element**	**Family**	**Atomic Fraction (%)**	**Atomic Error (%)**	**Mass Fraction (%)**	**Mass Error (%)**	**Fit Error (%)**
	6	C	K	53.89	3.69	43	3.45	11.43
	7	N	K	15.33	2.82	14.27	2.61	2.12
	8	O	K	12.66	2.39	13.46	2.48	1.56
	9	F	K	6.62	1.36	8.36	1.66	4.44
	11	Na	K	4.71	0.97	7.2	1.43	2.43
	12	Mg	K	1.7	0.37	2.75	0.58	4.75
	13	Al	K	0.31	0.08	0.55	0.14	16.5
	14	Si	K	1.52	0.32	2.84	0.58	4.63
	15	P	K	0.75	0.16	1.54	0.33	7.61
	16	S	K	0.71	0.15	1.52	0.32	7.52
	17	Cl	K	1.29	0.26	3.04	0.59	4.83
	19	K	K	0.12	0.05	0.31	0.12	33.97
	20	Ca	K	0.25	0.05	0.66	0.13	12.57
	26	Fe	K	0.14	0.04	0.52	0.14	21.51

**Area 7**	**Z**	**Element**	**Family**	**Atomic Fraction (%)**	**Atomic Error (%)**	**Mass Fraction (%)**	**Mass Error (%)**	**Fit Error (%)**

6	C	K	50.81	3.87	39.46	3.53	12.18
7	N	K	10.03	1.97	9.08	1.78	2.72
8	O	K	18.91	3.32	19.56	3.34	0.59
9	F	K	7.47	1.5	9.18	1.79	2.04
11	Na	K	4.8	0.99	7.13	1.42	1.54
12	Mg	K	2.03	0.43	3.19	0.66	1.91
13	Al	K	0.29	0.07	0.5	0.12	12.48
14	Si	K	1.5	0.31	2.73	0.55	2.43
15	P	K	1.51	0.31	3.02	0.6	3.29
16	S	K	0.61	0.13	1.27	0.26	4.43
17	Cl	K	1.53	0.3	3.52	0.67	2.09
19	K	K	0.17	0.04	0.42	0.09	12.04
20	Ca	K	0.29	0.05	0.74	0.12	5.46
26	Fe	K	0.05	0.02	0.19	0.06	28.7

Supplementary Table 3. The proteome data of *Nitrosopumilus maritimus* SCM1. See separate file in the supplemental data.
